# Reliability and Validity of Simplified Chinese Version of Roland-Morris Questionnaire in Evaluating Rural and Urban Patients with Low Back Pain

**DOI:** 10.1371/journal.pone.0030807

**Published:** 2012-01-27

**Authors:** Honglei Yi, Xinran Ji, Xianzhao Wei, Ziqiang Chen, Xinhui Wang, Xiaodong Zhu, Wei Zhang, Jiayu Chen, Diqing Zhang, Ming Li

**Affiliations:** 1 Department of Orthopaedic Surgery, The Affiliated Changhai Hospital of the Second Military Medical University, Shanghai, China; 2 No. 89 Hospital of PLA, Weifang, Shandong, China; University of Michigan, United States of America

## Abstract

**Objective:**

The causes of low back pain in China and Western countries are extremely different. We attempted to analyze the risk factors of low back pain in urban and rural patients under the dual economy with the simplified Chinese version of Roland-Morris disability questionnaire (SC-RMDQ) to demonstrate that SC-RMDQ could evaluate patients with low back pain arising from different causes.

**Methods:**

Roland-Morris disability questionnaire was translated into SCRMDQ according to international guidelines for questionnaire adaptation. In this study, causes of low back pain of 187 outpatients and inpatients (99 urban patients and 88 rural patients) were analyzed. All patients underwent simplified Chinese version of Roland-Morris disability questionnaire (SC-RMDQ), simplified Chinese Oswestry disability index (SCODI) and visual analogue scale (VAS). Reliability was tested using reproducibility (intraclass coefficient of correlation – ICC) and internal consistency (Cronbach's alpha). Validity was tested using Pearson correlation analysis.

**Results:**

The leading causes for low back pain were sedentariness (38.4%) and vibration (18.1%) in urban patients and waist bending (48.9%) and spraining (25%) in rural patients. Although causes of low back pain in the two groups of population were completely different, SCRMDQ had high internal consistency (Cronbach's α value of 0.874 in urban patients and 0.883 in rural patients) and good reproducibility (ICC value of .952 in urban patients and 0.949 in rural patients, P<0.01). SCRMDQ also showed significant correlation with Simplified Chinese version of Oswestry disability index (SCODI) and visual analogue scale (VAS) in rural areas (SCRMDQ-SCODI r = 0.841; SCRMDQ -VAS: r = 0.685, P<0.01) and in urban areas (SCRMDQ-SCODI: r = 0.818, P<0.01; SCRMDQ –VAS: r = 0.666, P<0.01).

**Conclusions:**

Although causes of low back pain are completely different in rural and urban patients, SCRMDQ has a good reliability and validity, which is a reliable clinical method to evaluate disability of rural and urban patients.

## Introduction

Low back pain is very common in clinical practice. More than 1/3 of patients visit orthopedic clinic due to low back pain [1]. In western developed countries, low back pain is very important for disability and industrial injury indemnification and 70%–80% of population ever suffered from low back pain with the prevalence of 30%. The relapse rate is very high and achieves 60–85% for patients with single low back pain history [2]. Low back pain is generally self-limited. A prospective study which was performed on 490,000 workers with low back pain in Sweden [3] showed that 57% recovered within one week, 90% within six weeks and 95% within 12 weeks. However, still 1.3% patients had disability after one year and need to be evaluated with a proper method such as Roland-Morris disability questionnaire (RMDQ) [4] and Oswestry disability index (ODI) [5] in western countries. RMDQ was firstly designed in 1983 by English scholars Roland and Morris to assess function of patients with low back pain and the contents were derived from 136-item sickness impact profile (SIP). SIP is a questionnaire reflecting general health status of patients. Roland et al. selected 24 items closely related with low back pain from SIP to compose RMDQ questionnaire and attached the premise “due to low back pain” in every item to distinguish disability arising from other causes. The score is 1 for every item. Answer “YES” gets 1 score and “NO” gets 0 score. All items have no difference in importance. The final score is defined as the sum of all scores with the minimum of 0 and the maximum of 24. A higher score is associated with more severe disability [4]. In recent years, RMDQ has been translated into more than 10 languages, including HongKong traditional Chinese version [6].

Traditional Chinese version of RMDQ serves for evaluation of low back pain and is consistent in content continuity, reliability and validity with original English version, Italy version, Japanese version and Tunisia version [7], [8]. However, due to differences in culture background and language, traditional Chinese version of RMDQ cannot be accurately understood and accepted by population in Chinese mainland. Incidence increases and causes of low back pain change significantly with rapid urbanization and industrialization as well as huge changes in life style and work environment in developing countries [9]. Especially in China, occupations, work positions, vibration, heavy thing-lifting types and rotation modes are extremely different from Western countries [9]–[11]. Therefore, the degree and risk factors of low back pain are also different. In this study, we tested the reliability and validity of simplified Chinese RMDQ (SCRMDQ) in evaluating disability of rural and urban patients with low back pain, attempted to demonstrate the evaluation accuracy of SCRMDQ of different causes and applicability in Chinese Mainland and further researched the treatment and prevention of low back pain in rural and urban patients in Chinese Mainland.

## Materials and Methods

### RMDQ translation

According to guidelines for questionnaire adaptation by Beaton [12], two Chinese spinal surgical specialists understanding English and one English professor without medical background translated the original RMDQ questionnaire into Chinese, independently. All specialists were blinded to the translation objective. Then, these translation texts were compared. The controversial or indefinite dictions were discussed and the original SCRMDQ was formed. After that, other two spinal surgical specialists and one English professor back translated the SCRMDQ into English version. The back-translating English version and original English version should achieve a consistency rate of 96%. Ambiguous contents in the SCRMDQ were corrected. At last, a specialist committee consisting of five spinal surgical specialists and one English professor discussed and revised the SCRMDQ for pilot trial. 44 outpatients with low back pain (30 women and 14 men with an average age of 45 year-old) filled in the SCRMDQ for pilot trial and were asked whether vague contents existed. The contents were revised until patients could get the right idea. For example, the term “change position” rather than “change posture” was completely understood by all patients. Thus, after proper modification, the finial SCRMDQ was established. The study was approved by the institutional review board of the Affiliated Changhai Hospital of the Second Military Medical University (Shanghai, China) and written informed consent was obtained from every participant.

### Subjects and methods

204 outpatients with low back pain in Changhai Hospital of Shanghai and No. 89 Military Hospital of China met the criteria, but 8.3% refused to fill in the questionnaire. A total of 187 patients completed the questionnaire, including 94 men and 93 women with an average age of 41.3±13.3 year-old (range18–79). Inclusion criteria were as follows: (1) low back pain had lasted for above half a month; (2) low back pain originated from spine which had been proved by a spinal surgical specialist; (3) the patient received at least 6-year compulsory education and accurately understood the questionnaire. Patients within the half a year after the surgery, fracture, cancer-related pain or drug abuse were excluded. All patients filled in the simplified Chinese SCODI 2.1 [13], visual analogue scale (VAS) and SCRMDQ. SCRMDQ was filled in by patient themselves and scores were computed. The original SCODI questionnaire included 10 items and every item had six alternative answers with the scores of 0–5. Concretely, scores of every item were summed up followed by dividing by the sum of 10-item highest scores (50 scores) and the resulting percentage was the actual SCODI score. VAS scoring was to rule a 100 mm straight line with the left end indicating “no pain” and the right end indicating “worst pain ever”. First, patients themselves drew dots in the line and the distance from the painless end to the dot was defined as the actual score. SCODI and VAS were also filled in by patients themselves and scores were computed. To evaluate the reproducibility of various questionnaires, two successive SCRMDQ, SCODI and VAS scores were compared using a rank sum test.

### Reliability test

To test reliability and consistency of simplified Chinese questionnaire in rural and urban population, internal consistency and test-retest reliability were assayed. Internal consistency was expressed as a Cronbach's α value [14] and test-retest reliability as a intraclass correlation coefficient (ICC). Test-retest reliability: 40 patients were randomly sampled from each group. SCRMDQ question sequence was broken to reduce the memory error. The two tests were conducted on an interval of 24 hours.

### Validity test

All patients were scored with SCRMDQ, SCODI and VAS on visit. Then, SCRMDQ was compared respectively with SCODI and VAS and correlation analysis was performed for consistency. An excellent consistency suggested this questionnaire was effective.

### Statistical analysis

SASS 11.0 was used for statistical analysis. Data were expressed as the mean ± standard deviation (SD) and were analyzed using a Pearson correlation method with the level of significance p<0.05.

## Results

A total of 187 patients, including 88 rural patients and 99 urban patients, completed the questionnaires. All patients came from two regions representing different economic development degrees: a general hospital in developed regions in eastern coast (Changhai Hospital) and a primary hospital in an inland underdeveloped region (the No. 89 hospital). All patients suffered from pain in duration from 28.9 months to 61.0 months ranging in ages from 18 to 79 years (Table 1). It was shown that the causes of low back pain were very different between rural and urban patients: waist bending (48.9%) and spraining (25%) for rural patients and sedentariness (38.4%) and vibration (18.1%) for urban patients (Table 2). Furthermore, table 3 also suggested that SCRMDQ scores were significantly higher in rural patients than urban patients, illustrating that varying occupations, work position and heavy thing-lifting types were correlated with scores, but Cronbach's α value of urban and rural patients were 0.874 and 0.883, respectively. The ICC value was 0.952 (0.909–0.975) in rural patients and 0.949 (0.903–0.973) in urban patients (p<0.01, Table 4). The correlation coefficient between SCRMDQ and SODI (0.841 in rural patients and 0.818 in urban patients) as well as between SCRMDQ and VSA (0.685 in rural patients and 0.666 in urban patients) showed an excellent consistency (Table 5). These results suggested that SCRMDQ had high reliability and validity in both rural and urban patients.

**Table 1:** Basic situation after the completion of the first questionnaire.

	Rural patients	Urban patients	Together
Sex: male/female	41/47	53/46	94/93
Age.	42.9±13.3 (18–71)	39.8±13.2 (18–79)	41.3±13.3 (18–79)
Pain lasting period (month)	32.7±74.0 (0.5–480)	25.4±46.7 (0.5–360)	28.9±61.0 (0.5–480)
Occupation			
Worker	29	19	48
Farmer	44	0	44
Officer	0	17	17
(white-collar) Businessman	5	15	20
Civil servant	5	9	14
Student	0	2	2
Retiree	5	18	23
Others		19	19

**Table 2:** The reasons for making low back pain in rural and urban patients.

	Rural patients (n = 88)	Urban patients (n = 99)	P value
Sedentariness	2 (2.3%)	38 (38.4%)	P<0.001
Waist bending	43 (48.9%)	13 (13.1%)	P<0.001
Standing fro long	1 (1.1%)	12 (12.1%)	P<0.003
Vibration	14 (15.9%)	18 (18.1%)	P = 0.702
Spraining	22 (25%)	5 (5.1%)	P<0.001
Others	6 (6.8%)	13 (13.1%)	P = 0.488

Different reason analysis of low back pain of rural vs. urban patients with Chi-squre, significant: p≤0.05.

**Table 3:** Scores of different questionnaires in rural and urban patients.

	Mean±SD	Range
SCRMDQ	7.6±5.8	0–24
Rural area	9.6±5.9	0–24
Urban area	5.8±5.1	0–24
SCODI	31.1±21.6	0–84
Rural area	37.6±23.1	0–84
Urban area	25.3±18.4	0–82
VAS	42.5±19.9	2–90
Rural area	46.1±20.3	3–90
Urban area	39.2±19.1	2–90

Questionnaire scores of rural vs. urban patients, p<0.05.

**Table 4:** SCRMDQ scores of two reproducibility tests (mean±SD, score, n = 40).

	Mean±SD		
	The first score	The second score	ICC
SCRMDQ (rural patients)	9.55±5.27	9.13±5.08	0.952 (0.909–0.975)
SCRMDQ (urban patients)	5.40±4.07	5.60±4.09	0.949 (0.903–0.973)

**Table 5:** Correlation analysis of validity of SCRMDQ with SCODI and VAS (Pearson correlation coefficient).

	r	P
Rural patients		
SCRMDQ-SCODI	0.841	P<0.01
SCRMDQ-VAS	0.685	P<0.01
Urban patients		
SCRMDQ-SCODI	0.818	P<0.01
SCRMDQ-VAS	0.666	P<0.01

## Discussion

Multiple function questionnaires evaluating low back pain are currently available, including RMDQ, ODI and VAS. RMDQ is widely applied to evaluate the functions of patients with low back pain since it is simple and easy to fill in and be followed up with the telephone and letter. There have been multiple RMDQ versions in various languages globally. However, causes and degrees of low back pain are different in rural and urban areas of China due to life and work conditions. Reliability of SCRMDQ and suitability to the two groups of population has not been reported.

Some studies illustrated that RMDQ had high reproducibility [9] in evaluating low back pain in rural patients, 0.91 in the same day, 0.88 on the interval of one week and 0.83 on the interval of three weeks. The study also proves that RMDQ has an excellent consistency with SF-36, the SIP and ODI. These illustrate that reproducibility and consistency are two important indices to evaluate the reliability of a questionnaire. In this study, the questionnaire is translated, back translated, modified and predicted in accordance with Beaton guidelines [12]. Patients come from two representative medical centers, a general hospital in developed regions in eastern coast and a primary hospital in an inland underdeveloped region. Due to imbalance of economic development in China, two-center studies can effectively prevent the bias from population in single center.

In this study, the Cronbach's α value of SCRMDQ is 0.883 in rural patients and 0.874 in urban patients, similar to recent studies, such as 0.94 for Tunisian version [7], 0.904 for Argentina version [15], 0.88 for Polan version [16] and 0.85 for Turkey version [17]. Retest reliability is 0.952 in rural patients and 0.949 in urban patients. The test interval is set as 24 hours, which is identical to that in the original English version and SCRMDQ question sequence is also changed in retest to reduce memory error. Results show a high satisfaction and are similar to Argentina version (0.94) [15] and Japanese version (0.95) [8]. In this study, SCRMDQ also shows a good consistency with SCODI and VAS both in rural patients with the correlation coefficient of 0.841 and 0.685. The correlation coefficients of SCRMDQ-SCODI and SCRMDQ-VAS in urban areas are 0.818 and 0.666, respectively. This study also indicates that SCRMDQ owns a good interval consistency, reliability and validity in evaluating low back pain in both rural and urban patients, is a reliable evaluation method and has excellent reproducibility and efficacy. In clinical practice, SCRMDQ can be used for self comparison and evaluation of disability inducing by low back pain before and after treatment and efficacy comparison of different treatment regimens to guide clinical treatment. In epidemiological survey on population with low back pain, SCRMDQ can serve as further evaluation for tested patients with low back pain to get knowledge on low back disability and guide intervention measures. Concurrently, this study also finds an interesting phenomenon, i.e. SCRMDQ scores have significant difference between rural and urban patients. It is reported that in China having the largest population, the annual incidence of low back pain is 50% in workers and teachers and 64% in farmers [18]. Moreover, severity of low back pain in rural patients is obviously higher than that of urban patients, primarily attributing to the fact that rural population is mainly occupied with agriculture and husbandry and long-term heavy physical labor and corresponding work position may accelerate low back pain. This indicates once more that low back pain is closely related with occupations and other factors. Therefore, the etiology of low back pain of urban and rural patients should be further investigated for target therapy, for example, patients due to heavy labor are treated with more rest and patients due to work position and life style are treated by changing position and enhancing physical exercise.

Although considerable epidemiological studies on low back pain have been conducted, the causes and pathogenesis are still unclear. Studies on low back pain are characterized by much subjective sensation, little objective positive test results and various and extensive risk factors. Therefore, animal model can not be copied completely. Epidemiologic studies are the major studying method. RMDQ is widely applied to understand the relationship between low back pain and its risk factors, to clarify pathogenesis of low back pain, and to investigate effective treatment and prevention methods. Now, multiple simplified versions are available such as RM-12, RM-16, RM-18, RM-23 and SIP-RM [19]–[21]. Some scholars suggest to modify RMDQ, for example, change the restrictive phrase “due to low back pain” to “due to low back pain or leg pain” for it is also applicable for patients with sciatica [22]. Stratford and Binkley [21] believed that the original questionnaire has some duplicate contents and can be simplified into 18 questions. Other scholars also raise some modification suggestions. However, because the original version is widely used in many countries, most specialists suggest to use the original version. In this study, SCRMDQ is the translation and verification version of the original version and has no modification. Evaluation results of low back pain with SCRMDQ are compared with that in different countries and regions. Reliability and validity tests are also performed. Thus, large-sample meta analysis on management of low back pain is possible. Therefore, we believe that SCRMDQ can be used for further study on affecting factors of rural and urban low back pain and pathogenesis.
